# Illness perception amongst adults with multimorbidity at primary care clinics in Southwest Nigeria

**DOI:** 10.4102/phcfm.v13i1.2738

**Published:** 2021-08-12

**Authors:** Babajide J. Ogunrinde, Adedotun A. Adetunji, Sufiyan A. Muyibi, Joshua O. Akinyemi

**Affiliations:** 1 Department of Family Medicine, University College Hospital, Ibadan, Oyo State, Nigeria; 2 Department of Epidemiology and Medical Statistics, University of Ibadan, Ibadan, Nigeria

**Keywords:** multimorbidity, illness perception, socio-demographic characteristics, Nigeria, primary care, chronic disease

## Abstract

**Background:**

Although shreds of evidence are emerging to show the role of illness perceptions in the health outcomes of patients, most of the previous studies have been on single chronic conditions.

**Aim:**

To assess the illness perceptions and the associated factors amongst adults with multimorbidity.

**Setting:**

General outpatient clinics of the University College Hospital, Ibadan, Nigeria.

**Methods:**

A cross-sectional study was conducted amongst a systematic sample of 403 adults with multimorbidity. Data on illness perception and other variables were collected using interviewer-administered questionnaires. Descriptive statistics, chi-square test, *t*-test and analysis of variance were employed for analyses.

**Results:**

The age of the participants ranged from 18 to 97 years, with a mean of 60.9 years (standard deviation [s.d.] ± 14.3 years). The majority of participants (57.3%) were women. Ninety-four (23.3%) respondents had only two morbid conditions, whilst 31.2% had at least four morbid conditions. Prioritisation sub-domain of illness perception recorded the highest score (mean = 2.0, s.d. ± 0.8), whilst the treatment burden sub-domain was the lowest (mean = 0.8, s.d. ± 0.7). A significant bivariate relationship was observed between emotional representation (*p* = 0.001), prioritisation (*p* = 0.013) and causal relationship (*p* = 0.013) sub-domains and age group of study participants. Emotional burden associated with illnesses declined as educational level increased (*p* = 0.039).

**Conclusion:**

Patient’s characteristics such as age, education and the number of morbidities are associated with illness perception. Healthcare providers should pay attention to these factors whilst addressing illness perception as a way to achieve better clinical outcomes.

## Introduction

Multimorbidity is defined as the co-existence of two or more chronic conditions, where one is not necessarily central than the others.^1^ The term ‘multimorbidity’ describes the presence of multiple health conditions in an individual. In defining chronic conditions for research purpose, O’Halloran found factors that are relevant to include: duration, prognosis, pattern and sequelae and diagnoses were based on the International Classification of Primary Care (ICPC)-2 classification system.^2^

There has been a gradual increase in the number of people suffering from multimorbidity driven by an increase in life expectancy and also by the growing burden of non-communicable diseases (NCDs). Life expectancy has been on the increase globally largely because of advances in medical sciences and technology turning formerly fatal acute diseases into survival events,^3^ which often results in chronic conditions and consequently increases people’s multimorbidity.^4^ Chronic conditions account for about 60% of all diseases worldwide and scientists projected an increase in the figure to 80% in 2020.^5^ It is estimated that about one-third of adult population lives with more than one chronic condition.^6^ In the developed countries, existing data suggest even a higher prevalence.^7^ Traditionally, developed countries have a high prevalence of NCD and consequently a higher rate of multimorbidity. In recent time, developing countries including Nigeria have been observing a change in the pattern of NCD. With the rise in the cases of NCDs, there is an emerging pattern of high levels of multimorbidity. The increase has been driven by urbanisation and changing lifestyles. Unfortunately, the socially and economically vulnerable people also face a risk of multimorbidity in Africa. The presence of multiple chronic conditions places extraordinary financial and psychological burden on the individual.

In recent years, evaluation of patients’ perceptions of illness is increasingly gaining recognition. Traditional biomedical approach to illnesses where diagnosis and treatment of a health problem are the only aims of healthcare is currently inadequate.^8^ Psychological adjustment to multimorbidity is particularly important because of its potential to affect the clinical, quality of life and cost outcomes.^9,10^ Illness perception is one of such psychological processes that can ensure adjustment to multimorbidity. Illness is the patient’s personal experience of sickness – the thoughts, feeling and altered behaviour of someone who feels sick.^11^ Croyle and Barger, quoted by Gibbons et al., defined ‘illness perception as beliefs about the cause, nature and management of illness, which enables patients to make sense of their conditions and better cope with the associated challenges’.^9^ They are an important predictor of how patients will behave during their illness experience and are directly associated with a number of health outcomes. The Common Sense Model of illness perceptions hypothesises that patients form both cognitive and emotional perceptions of illness and these organised beliefs about illness and treatment may influence quality of life. Patients with multimorbidity may demonstrate additional perceptions concerning multimorbidity itself, which may include perceptions of treatment burden from multimorbidity, causal relationships between conditions, priorities amongst conditions as well as synergies and antagonisms between conditions.^9^ Understanding illness perceptions is important because they can reliably predict health behaviours^9^ Evidence that interventions designed to modify illness perceptions can improve health outcomes such as health-related quality of life for patients with single conditions is emerging.^9^ Additionally, it has been suggested that illness perception model holds promise for understanding treatment decisions and adherence in adults with a single disease.^12^ However, research evaluating the clinical usefulness of illness perceptions in patients with multimorbidity is currently limited. Therefore, this study was conducted to assess the illness perceptions amongst adults with multimorbidity and the factors associated with it.

## Methodology

### Study design and setting

A cross-sectional study was carried out at the General Outpatient (GOP) clinic and Chief Tony Anenih Geriatric Centre (CTAGC) at the University College Hospital (UCH), Ibadan. Ibadan is the capital city of Oyo state, one of the 36 states in Nigeria. The bordering states of Oyo are Ogun state in the south, Osun state in the east and Kwara state in the north. Oyo state is located in the south western part of Nigeria, and has an estimated population of 5.59 million people, with about half of them (2.55 million) residing in Ibadan. Women constitute about 50% of the population of Ibadan according to the 2006 census was the most recent in Nigeria. The Yoruba ethnic group dominates Ibadan city.

The UCH is a tertiary academic institution with about 1000 beds located in Ibadan North, Local Government area of Oyo state. The University College Hospital, founded in 1957, was the first teaching hospital in Nigeria.

Patients from across Nigeria and the West-African sub-region are referred to UCH. The hospital provides tertiary care as well as primary and secondary care to residents of Ibadan and its neighbourhood because of an inefficient primary and secondary healthcare system in the region. The GOPs Clinic offers primary and secondary care, including emergency care, from where patients who require tertiary care are referred.

The GOP clinic attends to all adult patients (< 60 years). The clinic is run from 08:00 to 16:00 every weekday. The clinic attends to health issues of patients presenting with both differentiated and undifferentiated conditions. The services are provided by competent consultant family physicians and postgraduate resident doctors, with appropriate referral to other specialties, whilst all older patients aged 60 years and above are managed at the Chief Tony Anenih (CTA) Geriatric Centre. In both clinics, triaging of patients is done by the doctors and nursing staff such that emergency cases are attended to promptly, irrespective of the order of presentation. Follow-up patients who already had registered files with the clinic are seen in the outpatient clinics after dropping their cards for file retrieval by the health record staff.

### Study population

The study population consisted of adults aged 18 years and above, attending the GOP clinic and the CTA-Geriatric Clinic, UCH, Ibadan, Oyo state, Nigeria from May 2016 to July 2016 (60 working days). The study population was recruited from both the GOP and CTA Geriatric clinics. Participants were recruited from the two clinics to obtain a fairly uniform distribution of the participants across both clinics. O’Halloran et al.’s definition of chronicity as chronic conditions lasting at least six months was used to ascertain patients with chronic conditions, whilst having more than two chronic conditions was regarded as multimorbidity. Debilitated patients who could not give consent and pregnant women were excluded from the study.

### Sampling method

A total of 403 consenting respondents were recruited using systematic random sampling based on the sampling interval calculated by dividing the average total number of patients to be seen over the study period by the sample size. From the health records section, the estimated number of patients with multimorbidity projected to attend the Geriatric Clinic UCH was 2100, whilst it was 1500 for the General Outpatient Clinic over the same three months period. The addition of the estimated number of patients in both clinics resulted in 3600. The estimated sample size was proportionately allocated to the two clinics as follows:

$$\begin{array}{l} \text{GOP Clinic}-1500/3600*403=168\\ \left(\text{over a period of 3 months}\right) \end{array}$$ [Eqn 1]

$$\begin{array}{l} \text{CTA Geriatric Clinic}-2100/3600*403=235\\ \left(\text{over a period of 3 months}\right) \end{array}$$ [Eqn 2]

Using the above figures (168 for GOP and 235 for CTAGC), and dividing by 60 working days for the period of study, there were three recruitments for GOP and four recruitments for CTAGC per day and a sampling interval of 8. This was done until the required sample size was attained.

A simple random sampling was done daily by ballot amongst the first eight patients by an independent observer (the matrons in the clinics). Sampling was commenced from the patient who picked the ‘yes’ ballot and every eighth patient thereafter was recruited. The next patient was recruited if there was no consent given.

### Data collection

A pre-test was performed at the outpatient clinic of Agbeke Mercy Hospital, a public–private partnership clinic with UCH, Ibadan. The clinic is manned by consultant family physicians and senior registrars from the Department of Family Medicine. A randomly selected sample of 20 respondents, who fulfilled the inclusion criteria, were administered the questionnaire in order to validate the research instrument.

The eligible respondents for the main study were recruited daily in the waiting room of each clinic on each interview day. The purpose, content and implications of the study were explained to the participants. They were assured that their responses would be kept confidential; they were free to decide whether or not they wanted to participate and would not suffer any consequences if they chose not to do so. When consent for participation was granted, the respondent was taken to a consulting room where a more detailed nature of the study was explained and informed consent was then sought. The questionnaire was administered to all consenting respondents and an identification code was assigned to each respondent on the questionnaire.

Data collection was done by means of an interviewer-administered questionnaire. The instrument included sections on socio-demographic characteristics, information on multimorbidity and illness perception. Morbidity was measured based on simple counts of chronic health problems coexisting in the same individual. No standardised assessment of disease severity in the multimorbid conditions found was undertaken. Illness perception was assessed using the Multimorbidity Illness Perceptions Scale (MULTIPleS) questionnaire.^9^

### Multimorbidity Illness Perceptions Scale

The MULTIPleS questionnaire was developed to assess an individual’s illness perception of the multiple long-term conditions they experienced. The MULTIPleS scale is divided into five discrete domains of Emotional representations (emotional responses to both the illness [such as anxiety, depression and anger] and its outcomes, i.e. fear for future complications), Treatment burden (effort expended in operationalising treatments, navigating healthcare systems and managing relationship with healthcare providers), Prioritisation (relative importance of condition), Causal relationships (beliefs about the cause and relationship between the different conditions) and Activity restriction (impact of the conditions on daily activities). There are 22 items that consist of Treatment burden = Items 3, 5, 8, 9, 10, 14; Prioritisation = Items 1, 7, 12, 16; Causal relationships = Items 4, 11, 13; Activity Restriction = Items 2, 6, 15; and Emotional representations = Items 17, 18, 19, 20, 21, 22. Items 1–16 are scored on a four-point Likert-type scale of 0–3 (where 0 = strongly disagree, 1 = disagree, 2 = agree and 3 = strongly agree), whilst items 17–22 are scored on a six-point Likert-type scale of 0–5 (where 0 = strongly disagree, 1 = moderately disagree, 2 = disagree, 3 = agree, 4 = moderately agree and 5 = strongly agree), with a composite score calculated as the mean of each of the individual item scores within the subscales. Higher scores reflect strong agreement with the construct assessed by individual scale. Reliability testing of scales yielded a Cronbach’s alpha score of 0.88.

### Data analysis

At the end of the study, the administered questionnaires were sorted out and coded serially. Frequency tables were generated for variables after cleaning the data. Data were analysed using the Statistical Package for Social Sciences (SPSS) version 20. Categorical variables were summarised using frequency and percentages, whilst mean and standard deviation (s.d.) were computed for numerical variables. Illness perception domains were analysed using the two-sample *t*-test and analysis of variance (ANOVA). The two-sample *t*-test was employed to investigate the difference in mean values between two groups, whilst ANOVA was used for three or more groups. All analysis was done at 5% level of significance.

### Ethical considerations

Ethical approval was obtained from the University of Ibadan and the University College Hospital Institutional Review Board UI/UCH Ethics committee (reference number: UI/EC/14/0434). Permission was also granted by the Family Medicine Department and Chief Tony Anenih Geriatric Clinic, University College Hospital. Written informed consent was obtained from each study participant prior to data collection. The respondents either signed the consent form or thumb-printed depending on their level of literacy.

## Results

### Socio-demographic characteristics

A total of 403 patients who satisfied the inclusion criteria were studied. The socio-demographic characteristics of these respondents are shown in Table 1. Their age ranged from 18 years to 97 years, with a mean age of 60.9 years (s.d. ± 14.3 years). The majority of respondents (231; 57.3%) were women, with a female to male ratio of 1.3:1. Further details on their socio-demographic characteristics as presented in Table 1 show that more than half of the respondents (236; 58.6%) were at least 60 years old. The majority of them (332; 82.4%) were married, followed by widows and widowers (47; 11.7%). Of the respondents, 195 (48.4%) had post-secondary education, whilst 88 respondents (21.8%) had no formal education. The employment status shows that 182 respondents (45.2%) were currently unemployed and 158 of them (39.2%) engaged in self-employment.

**TABLE 1:** Socio-demographic characteristics of the respondents.

Variables	Male (*n* = 172)		Female (*n* = 231)		Total (*n* = 403)	
	*n*	%	*n*	%	*n*	%
**Age group (years)**						
< 30	5	2.9	4	1.7	9	2.2
30–39	13	7.6	12	5.2	25	6.2
40–49	29	16.9	23	10.0	52	12.9
50–59	29	16.9	52	22.5	81	20.1
60–69	37	21.5	71	30.7	108	26.8
≥ 70	59	34.2	69	29.9	128	31.8
**Marital status**						
Single	9	5.3	6	2.6	15	3.7
Married	154	89.5	178	77.1	332	82.4
Divorced/separated	4	2.3	5	2.2	9	2.2
Widowed	5	2.9	42	18.1	47	11.7
**Highest education**						
No formal education	15	8.7	73	31.6	88	21.8
Primary	10	5.8	28	12.1	38	9.4
Secondary	42	24.4	40	17.3	82	20.3
Post-secondary	105	61.1	90	39.0	195	48.5
**Employment status**						
Unemployed	80	46.5	102	44.2	182	45.2
Government employee	24	14.0	19	8.2	43	10.7
Non-government employee	10	5.8	10	4.3	20	5.0
Self-employed	58	33.7	100	43.3	158	39.1
**Religion**						
Traditional Religion	-	-	1	0.5	1	0.3
Christianity	121	70.3	150	64.9	271	67.2
Islam	51	29.7	80	34.6	131	32.5
**Ethnic group**						
Yoruba	159	92.4	212	91.8	371	92.1
Non-Yoruba	13	7.6	19	8.2	32	7.9

### Morbidity patterns

Table 2 shows that 103 (25.3%) respondents had only two morbid conditions, whilst 31.2% of them had at least four morbid conditions. Of all morbidities, the most common diagnoses included uncomplicated hypertension (69.0%), overweight (21.1%), obesity (32.9%), non-insulin-dependent diabetes mellitus (27.8%), osteoarthritis of the knee (27.0%) and back syndrome with non-radiating pain (22.1%).

**TABLE 2:** Common diagnoses in respondents.

Clinical findings (*n* = 403)	Frequency	%
**No. of morbid conditions**		
2	103	25.3
3	177	43.5
4+	127	31.2
**Diagnosis**		
Uncomplicated hypertension	281	69.0
Complicated hypertension	32	7.9
Back syndrome with radiating pain	36	8.8
Back syndrome with non-radiating pain	90	22.1
Osteoarthritis	110	27.0
Other forms of osteoarthritis	36	8.8
Insulin-dependent diabetes	3	0.7
Non-insulin-dependent diabetes	113	27.8
Overweight	86	21.1
Obesity	134	32.9
Lipid disorder	73	17.9
Cataract	49	12.0

### Morbidity according to age group of respondents

**FIGURE 1:**
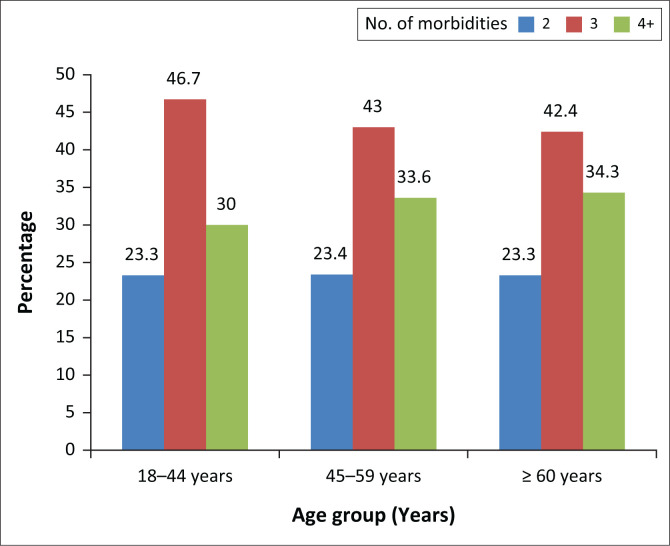
Number of morbidities according to respondents’ age group.

The number of four and more morbidities gradually increased with age as shown in Figure 1. The elderly age group (≥ 60 years) had more than 4 morbidities.

### Morbidity according to sex of respondents

**FIGURE 2:**
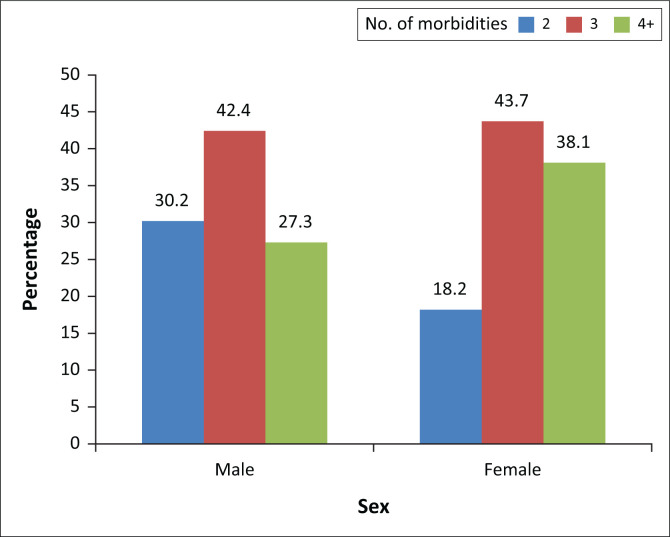
Number of morbidities according to sex of respondents.

Women recorded higher number of three or more morbidities as shown in Figure 2.

### Illness perception

Results on illness perception domains presented in Table 3 show that the prioritisation sub-domain recorded the highest score (mean = 2.0, s.d. ± 0.8), whilst the treatment burden sub-domain was lowest (mean = 0.8, s.d. ± 0.7).

**TABLE 3:** Descriptive statistics for illness perception domains.

Illness perception domains	Mean	s.d.
Emotional representation	1.3	1.2
Treatment burden	0.8	0.7
Prioritisation	2.0	0.8
Causal relationships	1.4	1.0
Activity restriction	1.3	0.9
Composite score	1.4	0.7

s.d., standard deviation.

### Association of illness perception domains with basic demographic variables and morbidities of respondents

Across the five sub-domains of illness perception in Table 4, there was no significant difference between men and women. However, a significant bivariate relationship was observed between emotional representation (*p* = 0.001), prioritisation (*p* = 0.013) and causal relationship (*p* = 0.013) sub-domains and age group of study participants. Emotional representation and prioritisation sub-domain scores were higher in the younger age group of 18–44 years and lowest in the elderly respondents (age ≥ 60 years). In contrast, scores for causal relationship followed a positive trend as it increased with age. Emotional representation was the only sub-domain of illness perception that statistically significantly (*p* = 0.039) varied by educational attainment such that the emotional burden associated with illnesses declined as educational level increased. None of the illness perception domains was related to type of employment. With regard to number of morbidities, a statistically significant bivariate relationship with illness perception was observed in the emotional representation domain, only with highest burden amongst those with at least four morbidities (*p* = 0.003).

**TABLE 4:** Association of illness perception domains with basic demographic variables and morbidity of respondents.

Patients’ characteristics	Emotional representation			Treatment burden			Prioritisation			Causal relationship			Activity restriction		
	Mean	s.d.	*p*-value	Mean	s.d.	*p*-value	Mean	s.d.	*p*-value	Mean	s.d.	*p*-value	Mean	s.d.	*p*-value
**Sex**			0.186			*0.404*			*0.21*			*0.24*			*0.484*
Male	1.23	1.24		0.77	0.63		1.95	0.83		1.37	1.05		1.35	0.92	
Female	1.40	1.31		0.82	0.67		2.06	0.84		1.49	1.01		1.28	0.96	
**Age group (years)**			0.001*			*0.058*			0.013*			0.013*			*0.423*
18–44	1.87	1.39		0.67	0.66		2.19	0.68		1.08	0.97		1.30	1.05	
45–59	1.29	1.21		0.73	0.67		2.02	0.92		1.46	0.94		1.21	0.98	
≥ 60	1.21	1.26		0.86	0.86		1.96	0.83		1.51	1.07		1.36	0.90	
**Education**			0.039*			*0.76*			*0.219*			*0.991*			*0.437*
None	1.35	1.29		0.86	0.59		2.01	0.81		1.44	1.01		1.27	0.96	
Primary	1.70	1.31		0.77	0.63		2.109	0.79		1.49	0.97		1.17	0.87	
Secondary	1.52	1.26		0.80	0.63		2.16	0.73		1.43	0.99		1.45	0.85	
Post-secondary	1.17	1.27		0.77	0.69		1.94	0.89		1.43	1.07		1.29	0.99	
**Employment status**			*0.404*			*0.51*			*0.996*			*0.686*			*0.063*
Unemployed	1.24	1.32		0.85	0.69		2.02	0.84		1.48	1.04		1.37	0.92	
Govt. employee	1.17	1.25		0.75	0.68		2.00	0.86		1.29	0.83		0.96	1.04	
Non-govt. employee	1.47	1.46		0.83	0.79		2.01	0.80		1.32	1.14		1.18	1.02	
Self-employed	1.44	1.23		0.75	0.58		2.03	0.81		1.42	1.04		1.35	0.92	
**No. of morbidities**			0.003*			*0.183*			*0.779*			*0.671*			*0.299*
2	1.25	1.26		0.79	0.73		2.04	0.86		1.36	1.08		1.24	0.99	
3	1.13	1.26		0.74	0.60		1.99	0.79		1.43	1.04		1.27	0.95	
4+	1.61	1.29		2.04	0.86		2.06	0.84		1.48	0.98		1.41	0.89	

s.d., standard deviation; govt., government.

* , *p* < 0.05, statistically significant.

## Discussion

This study aimed to assess illness perceptions amongst adults with multimorbidity and the factors associated with the illness perception. Prioritisation (relative importance of conditions) sub-domain of illness perception was observed to have the highest score. Other domains such as causal relationships, emotional representation and activity restriction scored higher than treatment burden sub-domain which had the lowest score. An exploratory study on illness representation of patients with multimorbidity amongst patients attending four selected general practitioners (GPs) practices in the United Kingdom reported higher representation in the perceived priorities and burden of medication amongst conditions.^13^ This is in contrast to this study that observed the lowest score in treatment burden. Treatment burden which refers to the effort expended in operationalising treatments, navigating healthcare systems and managing relations with healthcare providers was perceived as such possibly because polypharmacy is a common practice amongst the local patients as well as social support generally available in treatment care. In a cross-sectional study that evaluated prescription pattern and patients’ opinion on healthcare practices in selected primary healthcare facilities in Ibadan South-Western, Nigeria, polypharmacy was observed, along with satisfaction with medication cost, affordability and accessibility of primary healthcare to a patient’s residence.^14^

Amongst associated factors that have been examined, this study showed an association between age and illness perception in which younger patients had poorer illness perception probably because of the lack of experience in handling their illnesses. A significant bivariate relationship was observed between emotional representation, prioritisation and causal relationship sub-domains and age group of respondents. Emotional representation sub-domain that defines emotional responses to both the illness (such as anxiety, depression and anger) and its outcomes (i.e. fear for future complications) scored higher in the younger age group and lower in the elderly respondents. Younger respondents perceived more emotional burden of the chronic conditions. The elderly age group perhaps perceived chronic conditions as the norm rather than an exception with ageing. In contrast, scores for causal relationship followed a positive trend as it increased with age. In causal relationship, the understanding of the illness which includes the causes and how they are linked may increase over time as age advances.

This finding concurred with a few other studies that concluded on age as a demographic factor that influences a person’s illness perception.^12,15^ However, some other studies reported no significant association between age and illness perception.^16,17^ These differences may be a result of different methodologies used in the studies.

The findings of this study showed that emotional representation was the only sub-domain of illness perception that significantly varied by educational attainment such that the emotional burden associated with illnesses declined as educational levels increased. Educated people possibly have better social contacts from improved communication skills as well as ability to seek health information. This can reduce emotional burden attached to a chronic illness. Some other previous studies similarly reported a positive relationship between patients’ educational level and their illness perceptions.^18,19^ Aalto and colleagues noted that respondents with lower education levels tend to have weaker illness control and more severe perceived consequences, whilst Boonsatean observed that education enhanced the respondents’ ability to understand their conditions. On the other hand, some previous studies found no association between illness perception and educational levels.^17,20^ The difference in the results of these studies was probably because of the difference in the methods used to analyse illness perception. Whilst this study analysed illness perception according to individual sub-domains, the previous studies with contrast opinion were analysed by a total score.

With regard to a number of morbidities, a significant relationship with illness perception was observed in the emotional representation sub-domain only, with highest burden amongst those with at least four morbidities. The number of four or more morbidities observed in this study gradually increased with increasing age groups. This is expected because many chronic conditions present as age increases, with more morbidities occurring in the elderly age group. This is similar to what Fortin and colleagues found in a prevalence study of multimorbidity amongst adults in family practice where the mean number of chronic conditions increased significantly with age.^21^ However, worthy of note is the significant number of three morbidities observed in the younger age group. This further suggests that multimorbidity is not exclusive to the elderly population. The common morbidities observed in the study were hypertension, obesity, non-insulin-dependent diabetes mellitus, osteoarthritis of the knee, back syndrome with non-radiating pain, overweight and cataract. These chronic conditions are usually present in adults with increasing age. These were similar morbidities documented in previous studies in Family Medicine Clinic of UCH, Ibadan.^22,23^ Both studies documented the prevalent chronic conditions as hypertension, non-insulin-dependent diabetes mellitus, osteoarthritis and cataract. Similar trends were also observed in some multimorbidity studies elsewhere^3,24,25,26,27^ although with slight differences in the varieties of multimorbid conditions. This is possibly because of the ease of accessing primary care as well as higher life expectancy in the developed nations.

The higher prevalence of chronic illness in older age group particularly when it comes in multiples can understandably create significant emotional burden. Such perceptions of high emotional burden need to be put into consideration when evaluating individuals with multimorbidities.

In this study, illness perception was not significantly different between men and women. Although women scored higher in most of the domains of illness perception except in activity restriction, there was no statistically significant difference between men and women across the five sub-domains of illness perception in this study. A similar gender experience was observed in a study of illness perception in a chronic disease.^17^ Similarly, a study by Alsen and colleagues showed no significant difference when comparing the dimensions of illness perception between male and female patients after myocardial infarction.^28^ Nevertheless, another study showed contrasting results.^29^ The reason may be because of the larger sample size and unequal gender distribution used in the previous study compared to the current study. This study has its limitations. Firstly, it was a cross-sectional study. As such, conclusions especially on causality of the observed associations cannot be drawn. Secondly, the study was also hospital-based. Hence, the ability to generalise the findings is limited. However, the results can be used as baseline data for future research.

## Conclusion

Multimorbidity is on the increase globally and Africa is no exception. The traditional biomedical outcomes in patients with multimorbidity need to be complemented by measures that focus on patients’ concerns, like how illness and treatment affect their lives.

Illness perception is an important facet in managing patients with chronic diseases and especially when it comes in multiples in the same individual. Important socio-demographic characteristics that require consideration include age, education and the number of morbidity in the same individual. Healthcare providers should ensure more emphasis on improving illness perception in a way to achieve better clinical management outcomes.

The increasing trend of multimorbidity and the projection of future increase calls for more research particularly in sub-Saharan Africa to enhance knowledge for better care of this increasing population.
